# Tumor Microenvironment in Pancreatic Intraepithelial Neoplasia

**DOI:** 10.3390/cancers13246188

**Published:** 2021-12-08

**Authors:** Friederike V. Opitz, Lena Haeberle, Alexandra Daum, Irene Esposito

**Affiliations:** Institute of Pathology, Heinrich-Heine-University and University Hospital Duesseldorf, 40225 Düsseldorf, Germany; Friederike.Opitz@med.uni-duesseldorf.de (F.V.O.); LenaJulia.Haeberle@med.uni-duesseldorf.de (L.H.); Alexandra.Daum@med.uni-duesseldorf.de (A.D.)

**Keywords:** PDAC, PanIN, pancreatic cancer, tumor microenvironment

## Abstract

**Simple Summary:**

Pancreatic ductal adenocarcinoma (PDAC) is a very aggressive neoplasm with a poor survival rate. This is mainly due to late detection, which substantially limits therapy options. A better understanding of the early phases of pancreatic carcinogenesis is fundamental for improving patient prognosis in the future. In this article, we focused on the tumor microenvironment (TME), which provides the biological niche for the development of PDAC from its most common precursor lesions, PanIN (pancreatic intraepithelial neoplasias).

**Abstract:**

Pancreatic ductal adenocarcinoma (PDAC) is one of the most aggressive tumors with a poor prognosis. A characteristic of PDAC is the formation of an immunosuppressive tumor microenvironment (TME) that facilitates bypassing of the immune surveillance. The TME consists of a desmoplastic stroma, largely composed of cancer-associated fibroblasts (CAFs), immunosuppressive immune cells, immunoregulatory soluble factors, neural network cells, and endothelial cells with complex interactions. PDAC develops from various precursor lesions such as pancreatic intraepithelial neoplasia (PanIN), intraductal papillary mucinous neoplasms (IPMN), mucinous cystic neoplasms (MCN), and possibly, atypical flat lesions (AFL). In this review, we focus on the composition of the TME in PanINs to reveal detailed insights into the complex restructuring of the TME at early time points in PDAC progression and to explore ways of modifying the TME to slow or even halt tumor progression.

## 1. Introduction

Pancreatic ductal adenocarcinoma (PDAC) is one of the most aggressive malignant tumors with a 5-year survival rate of about 10% [1,2,3]. By 2030, PDAC is expected to be the second leading cause of cancer-related deaths in the United States. Currently, the main problem is late diagnosis and consequent poor prognosis with limited therapy options [4,5]. A hallmark of PDAC is the formation of an immunosuppressive tumor microenvironment (TME), leading to an evasion of immune surveillance. The TME is an assembly of desmoplastic stroma within the PDAC tissue, which is largely composed of cancer-associated fibroblasts (CAFs), immunosuppressive immune cells, immunoregulatory soluble factors, neuronal network cells, and endothelial cells [6,7,8,9] with complex mutual interactions [10,11,12,13,14,15]. It is known that PDAC can arise from different precursor lesions, for example, pancreatic intraepithelial neoplasia (PanIN), intraductal papillary mucinous neoplasms (IPMN), mucinous cystic neoplasms (MCN), and possibly, atypical flat lesions (AFL) [16]. PanINs are one of the best characterized and most frequent precursor lesions of PDAC. They consist of microscopic flat or papillary epithelial proliferations, typically in small pancreatic ducts. Depending on the degree of dysplasia, they display different amounts of mucin, differential architectural patterns, and variable proliferation rates [17,18,19,20,21]. In this review, we focused on the composition of the TME in PanINs to gain detailed insights into the complex restructuring of the TME at early time points of tumor progression and to explore the possibilities of acting on the TME to slow down or even arrest tumor progression.

## 2. Genetic Profile of PDAC and PanIN

To date, there are a large number of sequencing studies analyzing the alterations of the genome in PDAC patients. Interestingly, mutations may be responsible for altered activation of signaling pathways that lead to modified cell composition and further drive the development of the TME [22]. Results of many studies have shown that PDAC is a highly heterogeneous disease at the molecular level. Mutations in four different genes were found to be the main drivers: oncogenic *KRAS* mutations on one hand, and mutations of the tumor suppressor genes *TP53, CDKN2A,* and *SMAD4* on the other. Activating *KRAS* mutations are found in more than 90% of PDAC cases [23]. *KRAS* activation leads to increased cell proliferation, differentiation, survival, and migration of cancer cells [24]; upon mutation, the oncogenic RAS proteins are shifted into an active mode, leading to constitutive stimulation mainly of the mitogen-activated protein (MAP) kinase, Phosphoinositid-3-kinase (PI3K), and Ras-like guanine nucleotide exchange factors (RALGEF) pathways [25]. Activating *KRAS* mutations represent the earliest alterations of the genome in PDAC development and are present in more than 90% of low-grade PanINs [26]. Furthermore, in vivo studies in mice have shown that a direct consequence of activating *KRAS* mutations is the development of PanINs, but also that combination with mutations in tumor suppressor genes such as *TP53*, *CDKN2A*, and *SMAD4* is required for PDAC development [27,28,29]. In many tumors including PDAC, active oncogenic KRAS induces a stress response, leading to oncogene-induced senescence and loss of the p16ink4a protein encoded by CDKN2A [30,31]. Furthermore, it has been shown that a hypermethylation of the CDKN2A promoter with subsequent loss of function of CDKN2A is detectable in PanIN lesions [32]. Loss of function of *CDKN2A* results in loss of blockage of entry into the S-phase of the cell cycle [33]. In general, loss of function of the gene *TP53* is known to occur in more than 75% of tumors and results from missense mutations and loss of heterozygosity [34,35]. Mutation of *TP53* results in the inability to express specific genes that can promote cell cycle arrest or apoptosis in response to DNA damage or cellular stress [36]. In contrast to the activating *KRAS* mutations, which occur very early and initiate the development of PanINs, mutations in *TP53* are detectable only rarely in high-grade PanIN lesions [37]. Genetic alterations of *SMAD4* are also seldomly found in advanced PanIN lesions and represent the final step to complete tumor development [37,38]. *SMAD4* is responsible for the transforming growth factor (TGF)-β-dependent effect against proliferation. In a GEM (genetically engineered mouse) model for PDAC, it was shown that *SMAD4* mutations can lead to increased tumor development [29]. In addition, loss of *SMAD4* is associated with poorer patient survival [39]. In summary, activating *KRAS* mutations lead to the development of PanINs at an initial stage. Along with progression to high-grade PanIN, additional mutations may occur such as those affecting *CDKN2A, TP53*, and *SMAD4*, which are often observed in PDAC. Interestingly, a study revealed that oncogenic mutations such as those of *KRAS* are able to induce fibroblasts that, in turn, initiate altered signaling in the tumor cells, so called reciprocal signaling [22]. In line with this, a recently published study has shown that CAFs primarily secrete acidic fibroblast growth factor (FGF1), which in turn leads to MYC-dependent oncogenic activity in tumor cells. Specifically, FGF1 is responsible for CAF-dependent activation of AKT, leading to further secretion of factors by CAFs that stimulate activity of the AKT/GSK3β axis and enhance MYC protein stability [40]. Another study by Dey et al. showed that oncogenic *KRAS* is able to affect host cells by activation type I cytokine receptors via cytokine secretion from T_H_2 cells [41]. These studies demonstrate that there is a link between genetic alterations in tumor cells and the induction of an altered TME, which subsequently affects tumor development.

## 3. Cellular Compartments of the TME

### 3.1. Cancer-Associated Fibroblasts (CAF)

The most important structural component and cell population of the TME are the cancer-associated fibroblasts (CAFs). They are responsible for the stromal reaction including the occurrence of dense desmoplastic stroma in PDAC. It is known that the population of fibroblasts may account for up to 90% of the total tumor mass of pancreatic tumors [42]. In addition, CAFs are able to functionally interact with immune cells in several ways: secretion of soluble immune factors, direct cell–cell contact, mechanical stimuli, or metabolic crosstalk. Typically, CAFs express markers such as podoplanin (PDPN), α-smooth muscle actin (α-SMA), stromal cell-derived factor-1α, and fibroblast specific protein-1 [42,43,44]. In addition, CAFs are known to be activated by transforming growth factor β (TGF-β), tumor necrosis factor α (TNF-α), platelet-derived growth factor (PDGF) or interleukin (IL)-1, -6 or -10 [44]. Among these, TGF-β plays a controversial role and may act as a tumor promoter as well as suppressor by regulating tumor growth, differentiation, and immune cell functions [45,46]. CAFs can behave as cancer-promoting CAFs (pCAFs) or cancer-restraining CAFs (rCAFs) [47].

pCAFs: CAFs secrete stromal components such as collagen types I and III, fibronectin, and proteoglycans, leading to an increase in mechanical pressure in the extracellular matrix and to an inhibition of vascularization and promotion of cancer cell migration [42]. Furthermore, CAFs are involved in epithelial-to-mesenchymal transition (EMT), cancer invasion, metastasis, and angiogenesis [48,49]. Recently, three different subpopulations of CAFs have been identified in PDAC by single cell transcriptomics. These three subpopulations have distinct localizations and functional characteristics [43,50,51]. In detail, activated fibroblasts are located in close proximity to cancer cells and are contractile. For this reason, they have been termed myofibroblastic CAFs (myCAFs). Characteristically, myCAFs respond locally to high levels of TGF-β secreted by tumor cells. This leads to the induction of SMA and collagen genes. TGF-β is the best-characterized trigger of myCAFs. By upregulating the protein βig-h3 in stromal cells, it leads to direct suppression of CD8+ T cell activity and induces M2 polarization of tumor-associated macrophages (TAMs) [52]. Furthermore, myCAFs can synthesize collagens and other ECM molecules, contributing to the tumoral immune landscape and leading to tissue stiffness, decreased immune cell infiltration, and increased interstitial fluid pressure [8,53]. In detail, depletion of αSMA+ myCAFs leads to a reduction in Col1 in the tumor stroma in mice. This results in an accelerated development of PanIN and PDAC formation and decreased survival. This mechanism is facilitated by upregulation of SOX9 signaling in cancer cells, leading to secretion of the chemokine CXCL5 with subsequent recruitment of MDSCs and suppression of CD8+ T cells [54]. In a previous study, depletion of αSMA+ myCAFs in PanIN or PDAC mouse tissue led to the formation of undifferentiated tumors with poor prognosis [8]. Additionally, human PDAC tissues with fewer myCAFs display an increased number of immunosuppressive CD4+ FoxP3+ Tregs and these features are associated with poorer survival compared to patients with higher numbers of myCAFs [8]. The second subpopulation of CAFs are inflammatory CAFs (iCAFs), which exhibit immunomodulatory functions. Compared to myCAFs, they are located in stromal areas more distant from tumor cells. Their hallmark is the secretion of inflammatory cytokines such as IL-6, IL-1, IL-21, and LIF [55]. The transdifferentiation from CAFs to iCAFs is triggered by TLR4-mediated induction of IL-1β in tumor cells [56]. Interestingly, iCAFs promote polarization of M2 TAMs on one hand, and increase the number of myeloid-derived suppressor cells (MDSCs) in the tumor on the other, leading to a decrease in CD8+ cytotoxic T cells [56,57]. The third subpopulation is antigen-presenting CAFs (apCAFs), which represent a distinct subset of iCAFs. They express characteristic marker molecules such as MHCII, CD74, and SLPI and are able to activate CD4+ T cells through a MHCII-dependent manner.

rCAFs: Some studies suggest that the innate function of fibroblasts in every tissue of the body is the suppression and protection against tumorigenesis [58,59]. The study by Özdemir et al. showed that α-SMA is a marker for rCAFs, as the depletion of α-SMA+ cells led to enhanced tumor growth in a PDAC mouse model with an increase in Treg to promote antitumor immunity [8]. Interestingly, there are few studies on CAFs in PanIN. A study by Miyai et al. showed that in contrast to PDAC, which displays higher α-SMA expression, indicating the presence of pCAFs, rCAFs with meflin expression characterize the PanIN stroma. These findings led to the hypothesis that meflin-positive rCAFs arise around metaplastic or transformed cells in PanIN and show a decrease in meflin expression with a concomitant increase in α-SMA expression during cancer progression, resulting in behavior such as pCAFs [47]. Furthermore, meflin is known to bind to BMP-7, which counteracts the action of TGF-β preventing fibrosis [60].

Interestingly, in a study by Garcia et al., another fibroblast subpopulation was found to be present in both PanIN lesions and PDAC tissue. This fibroblast population upregulated the expression of Gli1 and was found in the progression to PDAC over PanIN lesions [61]. Gli1 is a target of the Sonic Hedgehog (SHH) signaling pathway and is activated in pancreatic CAF, thus promoting pancreatic cancer progression. Interestingly, the study by Steele et al. demonstrated that the SHH signaling pathway is enriched in the myCAF population in murine and human pancreatic cancer tissue. Furthermore, complete inhibition of Gli1 leads to depletion of the myCAFs and concomitant enrichment of iCAFs, resulting in a fibroinflammatory stroma [62].

In summary, there are currently only limited studies that have investigated the role of CAFs in precursor lesions, leading to controversial results.

### 3.2. Pancreatic Stellate Cells (PSC)

A specific subpopulation of CAFs are pancreatic stellate cells (PSCs) [44,63,64]. They were identified in 1998 and are a rare cell population in healthy pancreatic tissue [65,66]. Under homeostatic conditions, they are quiescent. Under inflammatory conditions or during carcinogenesis, they show specific histological and immunophenotypical changes characterized by star-shaped morphology, increased proliferation, deposition of ECM proteins, and expression of α-SMA [67]. Numerous studies have shown that PSCs can promote pancreatic cancer cells by increasing their proliferation and migration during standard culture conditions. This is mediated by the secretion of growth factors and cytokines by PSCs such as TGF-β or IL-6 [68]. Accordingly, the co-injection of PSCs together with tumor cells in an orthotropic mouse model of PDAC leads to increased tumor size and higher incidence of metastasis [69,70]. Some recently published in vitro studies have demonstrated that PSCs can differentiate into iCAFs or myCAFs by reprogramming of their differentiation program, which is dependent on the presence of the signaling molecules TGF-β and IL-1 [71,72]. Furthermore, in an in vivo KPC mouse model, PSCs were able to give rise to a minor subset of CAFs in PDAC tissue. This subset of CAFs was involved in modulating the TME by producing ECM components such as tenascin or perlecan [73]. A study by Nagathihalli et al. demonstrated that PSCs can actively secrete IL-6, leading to activation of the STAT3 signaling pathway in PanIN cells. This IL-6 secretion promotes the tumorigenic capacity of PanIN lesions [74]. Interestingly, one study demonstrated that there were fewer PSCs in early PanIN lesions than in late PanIN lesions and PDAC tissues [75]. Taken together, PSCs can already be identified in early PanIN lesions and show tumor-promoting properties by leading to the progression of high-grade PanIN lesions and, ultimately, to PDAC.

### 3.3. Immunosuppressive Cells

The innate immune system is the first line of defense against pathogens. In addition, these cells protect the body from malignant cells. The myeloid lineage includes granulocytes, macrophages, monocytes, and dendritic cells, and recognizes cancer cells by triggering antitumor responses and inflammation. Tumor cells can subvert this recognition by developing evasion mechanisms that become drivers of tumor progression in pancreatic cancer. Interestingly, myeloid cells play a dual role by initiating antitumor responses and promoting local inflammation, which can lead to chronic cancer-associated inflammation [76,77,78,79]. In PDAC, the immunosuppressive microenvironment consists of immunosuppressive tumor-associated macrophages (TAMs), myeloid-derived suppressor cells (MDSCs), regulatory T cells (Treg), and regulatory B cells (Breg), which are summarized in Table 1 [80,81,82,83,84].

#### 3.3.1. Tumor-Associated Macrophages (TAM)

**Figure 1:**
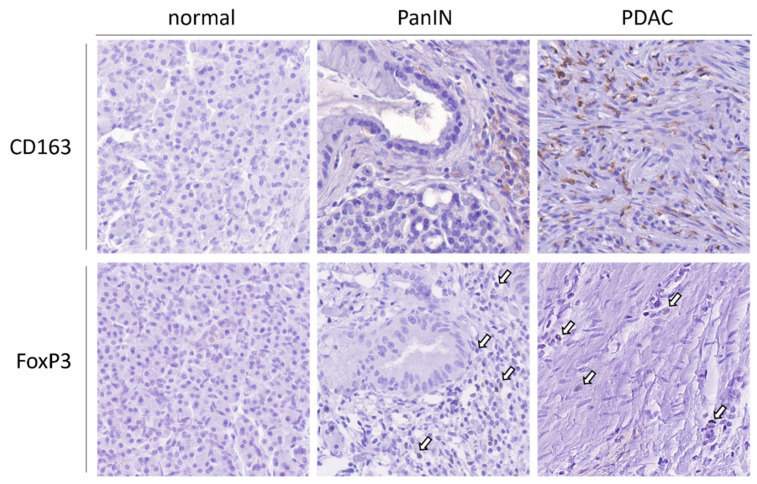
Immunosuppressive cells are rarely expressed in normal pancreatic tissue. In the stroma surrounding PanIN, M2 TAMs (CD163) and Treg (FoxP3) are rarely found. In contrast, cell numbers increase in PDAC tissue. Magnification 40×, arrows highlight FoxP3+ cells.

Macrophages are a heterogeneous population of cells with the majority of macrophages in healthy and inflamed tissues originating from the bone marrow. Furthermore, there are tissue-resident macrophages that are specialized populations such as alveolar macrophages in the lung, microglia in the brain, and Kupffer cells in the liver [85]. These cells can differentiate into tumor-associated macrophages (TAMs) in the presence of cytokines, chemokines, or growth factors such as GM-CSF, IL-3, CXCL12, CCL2, or other environmental factors such as local anoxia or high lactic acid concentrations [86,87]. In addition, TAMs can exhibit different polarization states, termed M1 and M2, during initiation, progression, and therapeutic intervention. Specifically, M1 macrophages represent cells with anti-neoplastic activity through the secretion of pro-inflammatory cytokines. In contrast, M2 macrophages enhance tumor progression and are characterized by the production of anti-inflammatory substances [88,89,90,91]. Many studies have demonstrated an association between patient prognosis and the presence of TAMs. Using PDAC mouse models, TAMs have been shown to be immunosuppressive and to promote angiogenesis, leading to tumor progression through the release of cytokines, chemokines, proteases, and growth factors [92,93,94,95]. To date, few studies have been published on the behavior of TAMs in PanIN lesions. The study by Pylayeva-Gupta was able to link the oncogenic Kras-induced production of GM-CSF in PanIN lesions to an immunosuppressive potential of Gr1+ CD11b+ myeloid cells [96]. Another study by Bastea et al. using an immunomodulatory agent to downregulate the M2 macrophage transcription factor interferon regulatory factor 4 showed that this resulted in reduced fibrosis in PanIN lesions and related tumors with concomitant activation of CD4+ and CD8+ T cells [97]. On the other hand, M1-polarized macrophages are known to enhance pancreatic cancer development through the contribution of acinar cell metaplasia. A study in mice showed that depletion of macrophages led to less development of acinar to ductal metaplasia (ADM) formation [98]. Furthermore, depletion of M1 macrophages leads to decreased ADM and PanIN formation. Macrophages are attracted by oncogenic *KRAS* in pancreatic acinar cells following upregulation of ICAM-1 [94]. A study by Liou et al. showed that IL-13 plays a critical role in the conversion of inflammatory macrophages into TAMs [99]. In summary, these studies could demonstrate that inflammatory macrophages as well as immunosuppressive M2 macrophages play a major role in the initiation of ADM, which in turn leads to the development of PanIN lesions (Figure 1).

#### 3.3.2. Myeloid-Derived Suppressor Cells (MDSC)

In general, myeloid-derived suppressor cells (MDSCs) form a heterogeneous immature myeloid cell population that is divided into two groups: granulocytic or polymorphonuclear (PMN-MDSC) and monocytic (M-MDSC) myeloid cells. PMN-MDSCs share the same phenotypic and morphologic characteristics as neutrophils, in contrast to M-MDSCs, which share these characteristics with monocytes. In TME, it has been shown that MDSCs revealed a strong increase, with PMN-MDSCs representing the majority of cells of all MDSC at more than 80% [100,101,102]. A correlation between clinical cancer stage and MDSC levels was observed in PDAC [103,104,105]. A mouse model suggests that tumor cells produce GM-CSF to stimulate recruitment and differentiation of MDSCs [96,106]. Furthermore, overexpression of the receptor RAGE was found in human PDAC, leading to an increase in the frequency of MDSCs and promoting carcinogenesis [107]. Proliferation of MDSCs in TME is driven by increased CD200 expression [108]. Another hallmark of MDSCs in pancreatic tumors is their suppressive nature for CD4+ and CD8+ T cells through direct cell–cell contact of MDSCs and lymphocytes [109] and stimulation of immunosuppressive regulatory T cells (Treg) through secretion of TGF-β and IFN-γ [110,111]. Little is known about the role of MDCSs in PanIN lesions, however, in agreement with the findings in PDAC, infiltrating MDSCs have been identified in early PanIN precursor lesions, not only in PDAC [112]. Interestingly, in a mouse study by Lesina et al., depletion of RelA in the pancreas was shown to lead to more rapid conversion of PanIN to PDAC by inducing MDSCs and blocking M1 macrophages [113]. This suggests that the RelA/CXCL1/CXCR2 axis is an important mechanism for tumor surveillance in PDAC. In the same lineage of senescence, Shimazaki et al. found in a secretome analysis of the PanIN and PDAC cell lines that complement factor B had an impact on the development of PDAC and is expressed in the TME, leading to accumulation of MDSCs [114]. Another study in mouse allografts found that immunosuppressive cell infiltration including MDSCs along with M2 macrophages leads to the formation of an immunosuppressive tumor microenvironment in precursor lesions as well as in PDAC tissues [115]. Furthermore, loss of type I collagen leads to Cxcl5-dependent recruitment of MDSCs with subsequent CD8+ T cell suppression during the course of development from PanIN to PDAC [54]. Taken together, little is known about the role of MDSCs in PanIN. However, there are some studies showing that MDSCs play a similar role in PanIN lesions as in the TME of PDAC tissues.

#### 3.3.3. Regulatory T Cells (Treg)

To date, the antitumor immune response is known to be downregulated in the complex pancreatic TME, with T cells in particular being exhausted in function [116]. An important mechanism for the balance between pro- and anti-tumor microenvironment is the regulation of CD4+ and CD8+ T cell populations. In particular, naïve CD4+ T cells are able to differentiate into Th1, Th2, Th17, Th22, and regulatory T cells (Treg). This mechanism is important for enhancing the effector T cell response. Furthermore, the immune system response is reflected by the ratio of Treg/Th17 [117,118]. The Treg subset is very important in maintaining self-tolerance by preventing excessive activation of T cells. This mechanism is a well-known defense strategy against autoimmunity. Interestingly, this defense strategy correlates with cancer progression [119,120]. Under normal conditions, secretion of inhibitory cytokines such as IL-10 or TGF-β by FoxP3+ Treg mediates suppression of effector T cells in the TME and results in anti-inflammatory properties and exhibits plasticity [121,122,123,124]. Notably, the pancreatic TME is composed of 25% Treg, which contributes to immunosuppression. In an in vivo mouse model, Tan et al. were able to correlate tumor regression with the disruption of Treg [125]. Another study demonstrated that depletion of Treg or blocking the TGF-β signaling pathway in a tumor mouse model of melanoma led to the prevention of immunosuppression of tumor-infiltrating CD8+ cells [126]. In contrast, the role of Th17 cells is still controversial. Th17 cells have been detected in human tumors and secrete IL-17, a potent cytokine for inducing inflammation by stimulating IL-6, TNF, chemokines, and matrix metalloproteases [117,127,128]. The presence of IL-17 and Th17 correlates with shorter overall survival, and higher amounts were found in tumor samples at a higher tumor stage [129,130]. These immunosuppressive cells are known to be present in precursor lesions of PDAC, particularly PanIN lesions, and lead to blockage of the antitumor activity of effector CD4+ and CD8+ T cells. This suggests that immunosuppressive cells play an important role in pancreatic tumorigenesis [112]. A study by Vizio et al. demonstrated that, on one hand, neutralization of IL-17 prevented the formation of PanIN and, on the other hand, forced IL-17 expression induced the development of PanIN into PDAC. Moreover, recruitment of Th17 to the TME was dependent on oncogenic KRAS expression in early PanIN lesions. In addition, autonomous expression of the IL-17 receptor was detected [131]. In this regard, there are two studies showing that infiltrating Th17 cells secrete the cytokine IL-17A, which leads to the development of PanIN via activation of the STAT3 signaling pathway. Specifically, pancreatic cells are able to express the IL17RA receptor after oncogenic *KRAS* activation, leading to downstream induction of REG3β. REG3β activates a signaling pathway dependent on gp130-, JAK2-, and STAT3-pathway that promotes cell growth. Furthermore, the formation of PanIN has been shown to be dependent on REG3β expression [132,133]. A mouse study by Keenan et al. demonstrated in KPC mice that depletion of Treg led to a decreased formation of early PanIN lesions [134]. In this direction, another study showed that SFRP4 expression increased in PanIN and PDAC tissue compared to normal tissue, and in addition, a positive correlation between Treg cell count and SFRP4 expression was found [135]. Thus, it is evident that Tregs are already present and active in PanIN precursor lesions and contribute to the development of PDAC (Figure 1).

#### 3.3.4. Regulatory B Cells (Breg)

Regulatory B cells (Breg) are a very newly defined and therefore not well-characterized cell population. In 2002, Mizoguchi et al. were able to designate B cells that contribute to immune tolerance and suppression of inflammation as Breg [136]. Furthermore, in some later studies, this Breg population was defined as a cell population that regulates disease development through various mechanisms such as the production of IL-10, IL-35, and IL-21 [137,138,139]. Interestingly, unlike T cells, any B cell subset has been described to differentiate into Breg. Only a stimulus of TLR ligands and anti-CD40 is required [140]. In addition, a study by Kalampokis et al. described that functional Breg can be induced by CD40 ligands, LPS, or CpG oligonucleotide stimulation [141]. In general, Breg are known to suppress immune responses against tumors, ultimately contributing to carcinogenesis. Specifically, some studies have shown that Breg produce IL-10 and TGF-β to suppress the antitumor effect of immune cells [142,143,144]. In addition, Olkhanud et al. demonstrated that Breg can promote the conversion of naïve T cells into Tregs by secreting TGF-β. Conversely, it is also possible that tumor cells are able to induce the conversion of normal B cells to Breg by inhibiting the antitumor immune process [145]. With regard to pancreatic neoplasms, one study investigated the role of B cells in PanIN and PDAC. B cells were found both in PanIN lesions and PDAC tissues of human and KPC mice and IL-35 was described as a major B-cell derived interleukin inducing tumor cell proliferation [84]. Consistent with this study, a recent study by Takhashi et al. found that IL-1β is a trigger for Breg activation through the IL-35 axis in a mouse model of PDAC and in human PDAC tissue [146]. Interestingly, a study by Das et al. demonstrated that treatment with Bruton’s tyrosine kinase (BTK) inhibitor of cytokine-induced B cells led to a decreased differentiation of Breg and production of IL-10 as well as IL-35. This BTK signaling pathway was also found in PanIN with an increase in cytotoxic T cells as well as tumor cell proliferation and PanIN growth. This implies that BKT is responsible for the regulation of Breg differentiation [147]. In summary, current knowledge suggests that Breg can also lead to an immunosuppressive tumor environment that promotes tumor growth. Furthermore, these milieu changes also play a significant role in early precursor lesions of PDAC.

### 3.4. Neuronal Cells

Neuronal cells are a further important component of the TME [148,149,150]. Perineural invasion is a frequent feature in PDAC [151]. In the TME, nerves contribute to the development of the vascular network that supplies oxygen and nutrients to the TME and removes excess metabolites [148]. Moreover, PSCs have been shown to express various neuronal proteins such as neurotrophins, which suggests the existence of crosstalk between stromal cells and neuronal cells [152]. Regarding the density of nerve fibers in PDAC, data are controversial: on one hand, one study showed that there was a correlation between low nerve fiber density and poorer survival [153]. Conversely, a study by Zhang et al. showed a correlation between increased nerve fiber density with tumor budding and poor survival [154]. Interestingly, a study by Saloman et al. demonstrated in their in vivo model that denervation in early stages of carcinogenesis such as PanIN lesions led to slower tumor progression and significantly prolonged survival [155]. Consistent with this, a study by Sinha et al. also showed that neurons promote proliferation of PanIN lesions by activating the STAT3 signaling pathway and that denervation in turn leads to loss of STAT3 and reduced PanIN formation [156]. In conclusion, neuronal cells play an active role in PDAC development.

## 4. Extracellular Matrix (ECM)

The extracellular matrix (ECM) is a three-dimensional non-cellular network composed of various molecules, which on one hand provide physical scaffolds and, on the other, can regulate processes such as growth, migration, differentiation, and homeostasis [157,158]. The ECM consists of various molecules, which interact with each other and are summarized in Table 2 [159]. The most investigated components of the ECM are structural and matricellular proteins such as Periostin (POSTN) and Tenascin (TNC). One of the hallmarks of PDAC is desmoplasia (cancer-associated fibrosis), which is histologically characterized by an abundance of ECM molecules [160,161,162], some of which are briefly reviewed here.

### 4.1. Proteoglycans

Proteoglycans are involved in altering the ECM during tumor formation through post-translational glycosylation [163]. In PDAC, the pancreatic TME is known to overproduce hyaluronic acid (HA). Interestingly, this overproduction begins early in carcinogenesis, and is present already in PanIN lesions [53]. A subgroup of proteoglycans are the heparan sulfate proteoglycans (HSPGs) associated with the cell surface or pericellular matrix. In this group transmembrane molecules, for example, glypicans as well as molecules that are secreted directly into the ECM such as perlecan exist [164]. Glypicans are located on the cell surface and anchored there with a C-terminal glycosylphosphatidylinositol-moiety [165]. In PDAC, Glypican-1 (GPC1) is highly expressed by cancer cells and CAF [166]. Furthermore, high GPC1 levels are associated with poorer differentiation and larger tumors [167]. Interestingly, there are some studies that have detected GPC1 in cancer cell exome sequencing studies [168]. In addition, GPC1 can be used as a marker for detecting early stages of pancreatic cancer such as PanIN [169]. One of the most important molecules of the basement membrane is perlecan. So far, perlecan has been shown to be a key molecule in the pro-metastatic environment. CAFs that are present in the stroma of metastases are known to be able to secrete high amounts of perlecan to attract cancer cells [164,170]. To date, there have been no studies addressing the role of perlecan in precursor lesions such as PanIN.

### 4.2. Collagen

**Figure 2:**
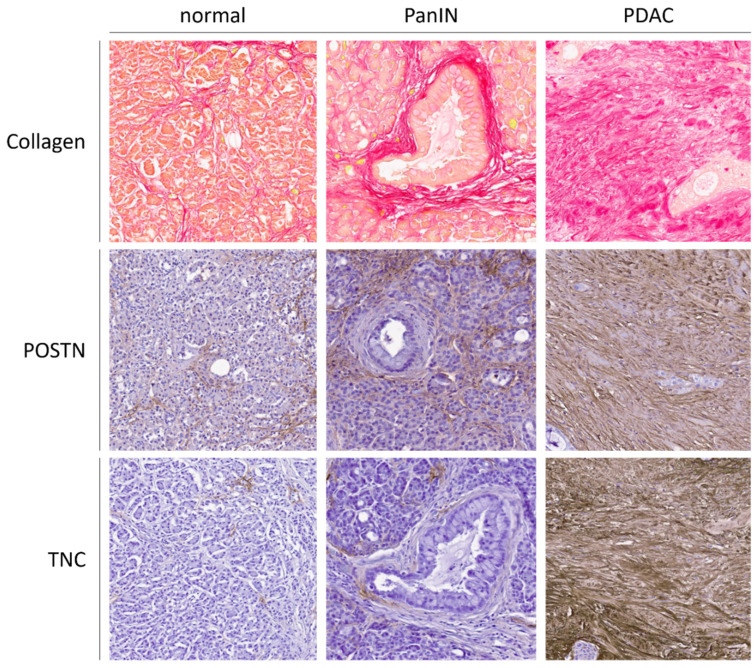
Proteins of the extracellular matrix (collagen, POSTN, and TNC) are expressed at low levels in normal pancreatic tissue. In the stroma surrounding the PanIN lesions, the expression of all three molecules increases and leads to a highly expressed, modified extracellular matrix composition. Collagen (red) was stained with Picro-Sirius red solution; POSTN and TNC were detected by immunohistochemistry. Magnification 20×.

The best-characterized structural ECM molecule in PDAC is collagen. To date, 28 different collagen types have been described [171]. Collagens can be divided into basement membrane collagens that include collagen IV, XV, and laminin [172], and interstitial collagens that include collagen I, III, and V [173]. Collagen I is known to be responsible for the desmoplastic reaction in PDAC [174,175,176,177]. With disruption of the normal architecture of the basement membrane, the exposure of PDAC cells to increasing amounts of interstitial collagen leads to the induction of protumorigenic effects. At the same time, high amounts of collagen I are associated with reduced patient survival [176,178,179]. Following the interstitial collagens, we demonstrated that collagen V is expressed by PSC and led to paracrine invasion and proliferation of the cancer cells and was also responsible for an enhanced metastatic potential. Moreover, collagen V was demonstrated to be highly expressed in PanINs [180]. Another important collagen associated with PDAC is collagen XV. Overexpression of collagen XV results in decreased migration of PDAC cells when cultured in collagen-rich matrices and is lost during pancreatic tumorigenesis [181]. On the other hand, it has been observed that the presence of collagen IV, which belongs to the same group as collagen XV and is an essential component of the basement membrane, correlates with dramatically decreased survival after resection of PDAC [182]. Furthermore, high expression of collagen IV in the stroma of PDAC leads to increased proliferation and migration of PDAC cells, moreover, these PDAC cells produce collagen IV themselves to protect themselves from apoptosis induced by serum deprivation. Not surprisingly, high levels of collagen IV in the serum may be associated with rapid relapse after surgery and poorer survival [183]. Collagen VI is highly expressed during PDAC development and is known to promote metastatic colonization, particularly in a hyperglycemic context [184,185]. In a study by Tian et al. [184], a comparison of normal pancreatic tissue, PanIN, and PDAC samples from mice and humans, was shown. During the progression of PDAC, at all levels of tumorigenesis, collagens are the most important group of proteins, accounting for more than 90% of ECM proteins at all stages. During this progression from normal pancreas to PDAC, fibrillary collagens such as COL1A1, 1A2, and 1A3 account for 90% of the total collagen mass. Interestingly, the above-mentioned study also showed that the complexity of the ECM increases during the progression of PDAC. In addition, 136 proteins were discovered to be overrepresented in the PanIN and PDAC samples compared with normal pancreas tissue, called the early PDAC progression signature. In summary, this implies that collagens are one of the most important molecules in ECM and are leading contributors to the development of fibrosis. Interestingly, the increased collagen accumulation can also be seen in the PanINs (Figure 2).

### 4.3. Periostin (POSTN)

Periostin (POSTN) is a matricellular multimodular protein composed of several subunits. In detail, it is composed of a signal peptide, a small cysteine-rich module, four fascilin-like domains, and a hydrophilic C-terminal region. These subunits have different functions. The signal peptide is necessary for secretion, while the small cysteine-rich module plays an important role in the formation of cysteine-disulfide bonds to form multimers. Fascilin-like domains are required for interaction with integrins, and the C-terminal region is responsible for interaction with other ECM molecules such as collagens or Tenascin C [186,187,188]. In normal tissues, POSTN is expressed in the periosteum and during embryonic development and body growth [189,190]. In addition, POSTN is expressed in connective tissues rich in collagen and in tissues subjected to mechanical stress such as the periosteum as well as during embryogenic development and body growth [191,192,193]. Through its ability to interact with cells via its FAS domains and with other ECM molecules via the N-terminal domain and C-terminal region, POSTN is able to act as a pro-survival protein in various cellular contexts [194,195,196]. Moreover, POSTN is known to play a key role in the cross-linking of collagen in the ECM [197,198]. In PDAC tissues, POSTN expression is strongly upregulated in cancer epithelial cells, PSCs, and stroma, and is associated with poor prognosis and worse tumor differentiation grade [184,199,200,201]. Furthermore, knockdown of POSTN in PSCs leads to a reduction in proliferation and metastasis of pancreatic cancer cells [202]. In the study by Erkan et al., POSTN was shown to be expressed at the invasive front of the tumor [203]. In a recent study by Yan et al., angiopoietin-like 4 was found to induce the formation of ductal cysts and was further responsible for silencing acinar genes and activating ductal genes, which is a hallmark of ADM and PanIN formation. Interestingly, POSTN acts as a downstream regulator of angiopoietin-like 4, and decreased ADM and PanIN formation in an angiopoietin-like 4-dependent manner in previous studies [204]. These studies demonstrate that an increase in POSTN expression is already present in PanIN and plays an important role in the onward progression to PDAC (Figure 2).

### 4.4. Fibronectin (FN)

Fibronectin (FN) is a glycoprotein that forms fibrils and is embedded in the extracellular matrix in all tissues. Its unique feature is that FN is composed of different domains, which allows this protein to interact with a variety of other molecules such as other ECM proteins or cell surface receptors [205]. The function of FN is mainly described as involvement in migration, cell adhesion, differentiation, and growth, which is an interesting hallmark of tumor development [206,207,208,209]. FN is known to be produced mainly by fibroblasts and to a lesser extent by cancer cells [210]. Moreover, cell–ECM interactions important for wound healing, tissue homeostasis, and development are supported by FN [211]. In contrast, the adhesion of FN to pancreatic cancer cells leads to the formation of a permissive environment that provides space for undisturbed proliferation of tumor cells. In this niche, tumor cells are protected from apoptosis, which helps tumor cells become chemoresistant [212]. Accordingly, there are studies showing that the expression of FN in cancer cells clinically correlates with poor prognosis and metastasis [213,214,215,216]. On the other hand, FN also shows tumor suppressive functions as FN loss of expression is correlated with malignant transformation [217,218]. In addition, FN is a widely used biomarker that can be detected at high levels in plasma, serum, or urine, indicating late, metastatic stages of cancer [219,220]. An example is the high expression of FN in circulating pancreatic tumor cells, which are known to have a high metastatic potential that is enhanced by the *WNT* pathway [221]. In general, the role of FN expression is controversial regarding its prognostic significance. However, studies by Leppänen et al. and Hu et al. demonstrated that FN expression is increased in PDAC tumor tissue compared with normal tissue, but is not associated with patient survival [222,223]. In contrast, studies by Hiroshima et al. and Javle et al. showed poor survival for patients with high FN expression associated with expression of p-ERK or ITGA3 [224,225]. For precursor lesions such as PanIN, a study by Dawson et al. demonstrated in a KPC mouse model that mice developed more PanIN lesions after a high-fat diet, which is, in turn, associated with increased FN expression [226]. Overall, FN expression is increased in PDAC tumor tissue, but the impact on overall survival is still controversial. In addition, the effects on precursor lesions such as PanIN have not been studied in sufficient depth.

### 4.5. Tenascin C (TNC)

The Tenascin family consists of four members, Tenascin C (TNC) being the best-characterized member and consisting of six monomers. These monomers are linked at their N-termini by disulfide bonds and form a hexamer. Thereby, each of the monomers showed different linearly arranged structural motifs with eight to 15 fibronectin repeats [227,228]. TNC is frequently expressed in embryonic tissue and in some adult tissue such as stem cell niches, but is mainly expressed de novo under pathophysiological conditions, especially during wound healing and tumor progression [229,230]. TNC is known to regulate the interactions between epithelial and stromal compartments. Both TNC and POSTN are members of the core matrisome, which is involved in the creation of the so-called metastatic niche of human neoplasms such as colorectal cancers, brain, and breast tumors [231,232,233]. Together, TNC and POSTN are able to form matrix networks, which leads to a synergistic metastasis reaction through the Wnt signaling pathway [186,229]. Furthermore, mouse studies using TNC-null mice and wild-type mice showed that TNC is involved in enhancing inflammatory responses [234,235]. In a mouse model for rheumatoid arthritis, TNC was responsible for activation of the toll-like receptor-4 (TLR4) pathway in macrophages and fibroblast, leading to the secretion of proinflammatory cytokines, and TNC has been shown to interact with immune cells and play a role in immunomodulatory effects [236]. Furthermore, TNC is known to be upregulated by TLR4 stimuli, which can be stored, leading to an autocrine loop in macrophages to trigger acute inflammation [237]. In addition, TNC can interact with various integrins through binding, which regulates adhesion, migration, and cell activation [238,239]. For PDAC tissues, TNC is known to reside in the tumor stroma and not in the tumor cells or normal pancreatic tissue, and the major source of TNC are PSCs [240]. One study showed that in PDAC, poor prognosis with high loco-regional recurrence rate correlated with a high perineural TNC expression [241]. Another recent study found TNC in the exosomal compartment, which is also associated with local invasion and metastasis [242]. A study by Leppänen et al. found high TNC expression in early-stage PDAC (T1-T2 tumors), which was associated with poor prognosis for patients [222]. An in vitro study by Paron et al. demonstrated that endogenous TNC promotes cell growth and migration in PDAC cell lines [243]. Interestingly, a very recent study by Barrera et al. showed that fibroblasts isolated and cultured from PDAC patients had higher expression of TNC with increased stromal activation. In contrast, depletion of TNC led to higher proliferation and migration of tumor cells, indicating an inhibitory effect of TNC on pancreatic tumor cells [244]. For early neoplastic lesions such as PanIN lesions, the study by Yoneura et al. demonstrated that the presence of TNC in PanIN and PDAC cells led to a morphological change to the mesenchymal phenotype in vitro [233]. We showed that the expression of TNC and of its binding partner annexin II on the cell surface is mainly present in PDAC tissue, but, interestingly, it shows an increasing progression from low-grade to high-grade PanIN and to cancer [240]. Furthermore, we could show that TGF-β1 is responsible for the TNC expression in PSCs. Another study by Zhang et al. showed that the RING-finger-containing protein RNF13 correlates with TNC expression and RNF13 is overexpressed in tumor samples. Interestingly, RNF13 expression was detected at an early stage in PanIN lesions [245]. Thus, TNC also appears to be detectable at the early stage of PanINs and to contribute to PDAC development at this time point (Figure 2).

## 5. Vascularization

Vascularization is a process in which capillary blood vessels grow or new vessels are formed. A hallmark during cancer development is the so-called angiogenic switch, in which a proangiogenic status is induced [246,247,248]. Interestingly, a study by Abdollahi et al. investigated angiogenesis by transcriptional analysis and demonstrated that there is an increasing gradient of angiogenic activation from normal pancreas tissue through precursor lesions to PDAC [249]. In addition, other studies have shown that tumor cells and associated endothelia express components of the VEGF pathway [250,251]. In contrast, there are studies showing that PDAC samples have lower microvessel density compared to the normal pancreas [252,253]. Other studies investigating the use of therapies against vascular mechanisms have failed. For example, the study by Kindler et al. showed that a monoclonal antibody against VEGFA did not improve survival in advanced pancreatic cancer [254]. Inhibition of the VEGF receptor also had no positive effect on patient survival [255]. Interestingly, a study by Zinczuk et al. demonstrated that the expression of carcinoembryonic antigen (CEA)-related cell adhesion molecules (CECAM) 1, CECAM 5, and CECAM 6, which are associated with angiogenesis, is upregulated in PanIN lesions and have been identified as an early marker in pancreatic carcinogenesis as it increases during development [256]. In addition, a study by Criscimanna et al. demonstrated that hypoxia plays an important role in PanIN lesions. They demonstrated that hypoxia inducible factor (HIF) 2α is expressed in early PanIN lesions and that HIF2α modulates Wnt-signaling during PanIN development [257]. Furthermore, the angiogenesis promoting molecule urocortin was shown to be more highly expressed in early PanIN lesions and well-differentiated PDAC [258]. Another study showed that neuropeptide Y receptor 2 (Y2) is significantly upregulated in both PanIN lesions and PDAC tissues und is thought to be responsible for modulating angiogenesis [259]. In summary, features of angiogenesis can be identified at early time points in PanINs and are present throughout the course of PDAC development. Thus, angiogenesis starts in the early phases of tumor formation before the invasive tumor is formed.

## 6. TME Targeting Strategies

TME targeting strategies in PDAC have been developed in the past years [260,261]. For example, matrix metalloproteinases (MMPs) have been used to suppress the development of the PDAC TME [262,263,264]. However, targeting MMPs has not been as successful as expected, possibly due to the complexity of the TME. Therefore, a number of alternative targets such as signaling pathways and specific cell populations are the subject of current studies. One controversially discussed target is the SHH pathway, as it is known to be a pro-tumoral signaling pathway that regulates crosstalk between stromal and tumor cells. In GEMMs, a study by Olive et al. demonstrated that the use of SHH inhibitors against smoothened (SMO) leads to the depletion of pancreatic tumor stroma and additional treatment with gemcitabine results in an increased number of apoptotic tumor cells and prolonged patient survival [252]. On the other hand, a recent study has shown that deletion of SMO in fibroblasts led to increased proliferation of tumor cells and proteasomal degradation of the tumor suppressor PTEN and was associated with poorer survival in PDAC patients [265].

Another strategy to influence the TME is stromal reprogramming. Stromal reprogramming aims to modulate the ECM structure (e.g., its density) to transform activated CAFs to a quiescent stage, or to normalize tumor vascularization. In the last five years, only a few studies have attempted to reprogram the stroma. A study by Laklai et al. found that a loss-of-function mutation in TGF-β signaling increased STAT3 activation with subsequent epithelial tension and contractility. In a next step, they demonstrated that stromal stiffening can be partially reversed by depletion or inhibition of STAT3 [266]. Furthermore, other studies have shown in mouse experiments that inhibition of STAT3 leads to remodeling of the stroma by decreasing the number of activated CAFs, leading to an improvement in the response to gemcitabine therapy [267,268].

Since the TME is characterized by tremendous infiltration of immune cells, reprogramming of cancer-inducing immune cells would be another promising target. As described above, TAMs and Treg are leaders in tumor development in PDAC. Therefore, targeting these immune cells may be a hopeful strategy. There are studies showing that inhibition of the recruitment axis of myeloid cells by the combination of chemokine receptor blockage and chemotherapy (CCR2 inhibitor/FOLFILINOX) leads to a decrease in infiltrating TAMs and Treg with an additional increase in CD4+ and CD8+ cells [269]. Furthermore, this combination prolonged the survival of mice bearing orthotopic KPC tumors [93].

To date, there have been few studies addressing TME targeting in PanIN lesions. A recent mouse study demonstrated that inhibition of Bruton’s tyrosine kinase with tirabrutinib resulted in an impaired Breg population with increased cytotoxic T cells and attenuated PanIN growth [147]. In another study, treatment with the immunomodulator pomalidomide was shown to lead to an absence of TAMs and a subsequent increase in CD4+ and CD8+ T cells, resulting in reduced fibrosis in PanIN lesions [97].

## 7. Conclusions

The TME is a complex network composed of many different components. Current knowledge suggests that a modified TME is already present around PDAC precursors, possibly playing a role in PDAC development. In the initial stage, mutations such as the activating *KRAS* mutation seem to play a key role. These alterations are already found in early precursor lesions such as PanIN, following inactivation of tumor suppressor genes such as CDKN2A, TP53m or SMAD4, which in turn contribute to an enhancement of the tumor stroma. Activated stromal cells such as PSC have already been described in low-grade PanIN, and their number increases during progression to PDAC. PSC are, at least partially, the source of collagens and of matricellular proteins such as POSTN and TNC, which exert important functions by affecting epithelial cell and immune cell properties. Immunosuppressive cells such as M2 macrophages, MDSCs, Treg, and Breg are important for tumor development because they promote evasion mechanisms that allow tumor progression. Immune cells are already present in the stroma of PanIN lesions and increase their number and effectiveness during development into PDAC.

In summary, relevant pro-tumorigenic changes in the microenvironment occurs very early in the process of PDAC progression. Further research should be directed to functionally investigate TME components in PDAC precursors to exploit their possible role as therapeutic targets to prevent progression to invasive cancer.

## Figures and Tables

**Table 1:** Summary of immunosuppressive cells that play a role during the development of PanIN precursor lesions and their functions.

Immunosuppressive Cells	Function in PanIN Lesions
TAMs	
M1	Induction of PanIN lesion formation through activation of oncogenic KRAS
M2	Activation of IRF4 induces fibrosis in PanIN lesions
MDSCs	Recruitment of MDSCs through the chemokine Cxcl-5 in PanIN lesions with subsequent CD8+ T cell suppression
Treg	Present in PanIN lesions, lead to blockage of effector CD4+ and CD8+ T cells Secretion of IL-17 enhances PanIN formation
Breg	Activated by IL-10 and IL-35, stimulate tumor cell proliferation in PanIN lesions

Breg: Regulatory B cells, CD: Cluster of differentiation, Cxcl-5: C-X-C motif chemokine 5, IL: Interleukin, IRF4: Interferon regulatory factor 4, KRAS: Kirsten rat sarcoma, M1: M1 macrophages, M2: M2 macrophages, MDSCs: Myeloid-derived suppressor cells, PanIN: Pancreatic intraepithelial neoplasm, TAMs: Tumor-associated macrophages, Treg: Regulatory T cells.

**Table 2:** Main components of the ECM with their specific function and examples.

Components of ECM	Function	Examples
Proteoglycans	Most important structural and functional biomacromolecules, which can interact with growth factors, cytokines, cell surface receptors and other ECM molecules	Heparan sulfate proteoglycans
Collagens	Fibrous proteins, which represent 30% of the total mass of proteins in humans; synthesized and secreted in the ECM by fibroblasts	Collagen I, Collagen III, Collagen V
Elastin	Large, very stable ECM structures enabling tissue recoil	Topoelastin
Fibronectin	Expressed by various cell types in the ECM and responsible for development in vertebrates; can interact with integrin receptors	Cellular fibronectin, plasma fibronectin
Laminin	Plays role in early embryonic development and organogenesis	Laminin 2, Laminin 5
Matricellular Proteins	Can facilitate cell-cell and cell-ECM interactions; promote cell adhesion and cell migration; show moderate expression in adult tissue, but increase under pathological conditions	Periostin, Tenascin

## Data Availability

No primary data were generated in the context of this work, as the nature of the work is a literature review.
